# Entrapment of the Median Nerve Following Elbow Dislocation in Children: A Case Series

**DOI:** 10.3390/medicina62101876

**Published:** 2026-09-28

**Authors:** Nathaniel F. R. Huang, Kemble K. Wang, Daniel J. Wilks, Danielle Nizzero, Erich Rutz, Jason Harvey

**Affiliations:** 1 Department of Orthopaedics, Monash Health, Dandenong, VIC 3175, Australiajharvey@osv.com.au (J.H.); 2 Department of Orthopaedics, The Royal Children’s Hospital, Parkville, VIC 3052, Australia; erich.rutz@rch.org.au; 3 Murdoch Children’s Research Institute, Parkville, VIC 3052, Australia; daniel.wilks@rch.org.au; 4 Department of Surgery, Monash University, Clayton, VIC 3800, Australia; 5 Department of Plastic Surgery, The Royal Children’s Hospital, Parkville, VIC 3052, Australia; danielle.nizzero@rch.org.au; 6 Department of Paediatrics, The University of Melbourne, Parkville, VIC 3010, Australia; 7 Department of Plastic Surgery, Monash Health, Dandenong, VIC 3175, Australia; 8 Department of Orthopaedics, Bob Dickens Orthopaedic Centre for Education and Research, The University of Melbourne, Parkville, VIC 3010, Australia; 9 Medical Faculty, University of Basel, 4001 Basel, Switzerland; 10 School of Health and Biomedical Sciences, Royal Melbourne Institute of Technology, Melbourne, VIC 3000, Australia

**Keywords:** median nerve entrapment, elbow dislocation, medial epicondyle fracture, paediatric, nerve injury

## Abstract

*Background and Objectives*: Median nerve entrapment following elbow dislocation is a very rare but serious condition in children. We describe four cases of median nerve entrapment, highlight diagnostic challenges, evaluate the impact of delayed treatment, and propose a treatment algorithm. *Materials and Methods*: A retrospective review of a consecutive case series of four children with median nerve entrapment following elbow dislocation was conducted. Clinical records, imaging, operative findings, and outcomes were reviewed. *Results*: In all patients, the causative injury was posterolateral elbow dislocation with an associated medial epicondyle fracture. Three patients had delayed diagnosis of their median nerve entrapment (at 10–31 months post-injury) and required nerve resection with sural nerve grafting; none achieved full neurological recovery. One patient underwent early exploration and decompression (at 4 days post-injury) and made a full recovery. Misattribution of neurological symptoms to transient neurapraxia, under-recognition of radiographic signs, and inadequate early advanced imaging contributed to delayed diagnosis and management. Magnetic resonance imaging was the most useful imaging modality for diagnosing median nerve entrapment, though initial reporting missed entrapment in one case. *Conclusions*: Prompt recognition and early surgical decompression are critical for optimal recovery in paediatric median nerve entrapment after elbow dislocation. Delayed treatment is associated with poor outcomes. We propose a treatment algorithm to guide management and improve outcomes.

## 1. Introduction

Elbow dislocations are the most frequent large-joint dislocation encountered in children [1,2]. Neurological injuries are reported in up to one-fifth of these cases, with the ulnar nerve more frequently involved than the median or radial nerves [3,4,5,6]. Intra-articular median nerve entrapment following elbow dislocation is a rare complication described in paediatric and adolescent populations [7,8].

Median nerve entrapment following elbow dislocation is associated with medial epicondyle fractures, as the avulsed medial epicondylar growth plate creates a path for the nerve to be entrapped in the joint or fracture site [2,8,9]. Fourrier, and later Al-Qattan et al., have categorised the injury into four types based on entrapment patterns [10,11].

Clinical diagnosis of median nerve entrapment is often delayed; in a recent literature review, 38 of 41 cases had a delay in diagnosis [7]. Assessing children can be challenging, and mild median nerve deficits may be overlooked or attributed to neurapraxia [2,3,4].

Matev [12] and Packer [13] describe a pathognomonic radiological finding of medial supracondylar ridge ossification associated with a radiolucent lumen from the entrapped nerve, which may alert the clinician to the diagnosis. Ultrasound (US) and magnetic resonance imaging (MRI) may also assist diagnosis [14,15,16,17,18].

If unrecognised, this injury is potentially catastrophic, leading to irreversible neurological deficit. No cases of full nerve recovery have been reported when diagnosis is delayed more than 4 months [7].

The literature describes fewer than 50 cases [7,8], most of which are isolated case reports or small series [12,13,14,15,17,18,19,20,21,22,23,24,25]. We present a case series of four children with intra-articular or intraosseous median nerve entrapment following elbow dislocation. We aim to highlight diagnostic challenges, the impact of delayed intervention on outcomes, and propose a treatment algorithm.

## 2. Case Series

Between 2014 and 2021, 4 consecutive children presenting to two tertiary paediatric hospitals with median nerve entrapment following elbow dislocation were included in the study. Medical records, operative reports, and imaging studies were reviewed retrospectively to assess injury characteristics, diagnostic work-up, intraoperative findings, surgical management, and outcomes. Functional outcomes were assessed based on recovery of motor and sensory function. Due to small patient numbers, no statistical analysis was possible.

## 3. Results

A summary of the four patients is provided in Table 1.

### 3.1. Presentation

Patients were aged between 9 and 13 years (average age 10.5 years). All patients sustained a posterolateral elbow dislocation with an associated medial epicondyle fracture. All patients underwent a closed reduction under sedation in an emergency department on the day of injury. Three patients had documented signs of nerve injury at presentation and immediately post elbow reduction. Their documented examination findings ranged from median nerve distribution paraesthesia to global hand paraesthesia with anterior interosseous nerve motor palsy, all of which were attributed to neurapraxia from the dislocation. All patients were placed in above-elbow plaster cast immobilisation post reduction. The three patients who had delayed diagnosis of their median nerve entrapment had their casts removed between 3 and 5 weeks post-injury.

Only one patient was diagnosed early with median nerve entrapment at day 4 post-injury. After initially presenting with median nerve paraesthesia and no documented motor deficit, she was reviewed as an outpatient at day 4 post-injury, by which time she had developed a dense median nerve motor palsy. The remaining patients had a delayed diagnosis of between 10 and 31 months. One patient likely had initially missed neurological symptoms, with a delayed diagnosis 2.5 years later of median nerve palsy. No concomitant vascular injuries were observed.

All patients underwent pre- (Figure 1) and post-reduction (Figure 2) radiographs in the emergency department, showing concentrically reduced joints and non-displaced medial epicondyle fractures. The three patients who had delayed diagnosis of their median nerve entrapment all had radiographic evidence of an entrapped median nerve present at the time of subsequent outpatient clinic follow-up that was not initially recognised by the treating teams (Figure 3). The radiographic findings were abnormal medial supracondylar ridge ossification with a radiolucent lumen, as described by Matev [12] and Packer [13].

Three patients underwent ultrasound, with two showing an abnormally positioned median nerve. All patients underwent MRI; in the three patients with delayed diagnosis, the decision for MRI was made to investigate chronic neurological deficits post-injury. In the case of the acutely diagnosed patient, the MRI was requested after the patient presented to outpatients at 4 days post-injury with a dense median nerve palsy. All MRIs demonstrated median nerve entrapment (Figure 4), although one was initially misreported as normal. In two patients, MRI showed median nerve entrapment between the medial epicondyle and the distal humerus; in the remaining patients, the median nerve was entrapped in the joint, behind the medial epicondyle.

### 3.2. Treatment and Outcomes

The one patient with early diagnosis of median nerve entrapment at day 4 underwent exploration and decompression of the median nerve, with fixation of the medial epicondyle. This patient regained full motor and sensory function of the median nerve by 9 months post-injury. The patient also regained full range of motion, with return to pre-injury sports.

The three remaining patients with delayed diagnosis all underwent surgical exploration, which confirmed an entrapped median nerve (Figure 5). One patient underwent nerve resection with sural nerve grafting at the time of initial exploratory surgery. The remaining two underwent decompression alone, with subsequent grafting at a later date after no meaningful nerve recovery was observed. All three of these patients achieved only partial recovery of their median nerve function. Median nerve innervated muscle power grading and sensation for each patient at the latest follow-up are detailed in Table 1. One patient (case 1) has persistent neuropathic pain. At the time of this review, one patient (case 3) is being considered for a brachioradialis to flexor pollicis longus tendon transfer. Patients had an average follow-up of 29 months (range 9–66 months).

## 4. Discussion

### 4.1. Discussion

Nerve injury following elbow dislocation is reported to occur in 5% to 22% of patients [2,3,19,23]. The ulnar nerve is the most commonly injured nerve, followed by the median and radial nerves [3,4,5,6]. The median nerve has been reported to be injured in up to 3% of elbow dislocations [3], and median nerve entrapment following paediatric elbow dislocation is a rare but well-documented complication. It was first reported by Gurdjian and Smathers in 1945 in a 7-year-old girl post elbow dislocation [26]. Fewer than 50 cases have subsequently been described in the English-language literature [7,8]. This series of four patients highlights the clinical and radiological challenges in diagnosing this rare condition, and the importance of early intervention to achieve good patient outcomes.

Early diagnosis appears to be the most significant challenge. In this series, three of four patients had delayed recognition of median nerve entrapment until many months following their injuries. Inadequate neurological examination or documentation and the tendency to attribute early symptoms to transient neurapraxia contributed to these delays. This is consistent with the reported literature, with delayed diagnosis of 1 month or more occurring in 85% of patients [7,8].

Other authors have previously highlighted the challenges of diagnosing a median nerve entrapment. The early clinical signs can be difficult to elicit in a frightened child with a painful dislocated elbow, as a thorough neurologic exam can be challenging, and mild median nerve deficits may be overlooked [2,3,4].

Neurapraxic injuries, which often present as incomplete loss of function and are, by definition, transient, are relatively common with paediatric elbow injuries [27,28]. The expectation by the treating clinician that the nerve injury is praxic may lead to prolonged hopeful observation when the correct approach is to investigate and intervene early.

We acknowledge that examination of the acutely painful child can be difficult, and false negative and false positive physical examination results are not uncommon. We recommend thorough neurological examination before and after dislocation reduction, as well as re-examination of the child at every follow-up point to maximise opportunity to detect neurological deficits. If uncertainty regarding nerve function is present, careful re-examination by a paediatric hand therapist can also be considered to maximise diagnostic reliability. The minimum age of children in our series is 9, and we believe that once acute anxiety following the initial trauma has passed, a good neurological examination is possible in most cases.

In this series, two patients returned to the emergency department within 24 h of injury reporting neuropathic pain in the elbow or forearm, which retrospectively may have served as a red flag. Ho et al. recommend surgical exploration for any new-onset or worsening median nerve signs following reduction [4]. Other clinical signs have also been proposed as possible red flags for nerve entrapment. In their case report of two children, Floyd et al. suggested that the combination of a proximal median nerve deficit, limited passive elbow motion, and an associated medial epicondyle avulsion fracture following a paediatric elbow dislocation should raise concern for possible entrapment [25].

All patients in this series had elbow dislocations with medial epicondyle fractures. The association between this injury pattern and median nerve entrapment has been previously described, and it is postulated that the avulsed medial epicondylar growth plate creates a path for the nerve to displace posteriorly and traverse the ulnohumeral joint [2,8,9]. Median nerve entrapment has also been described in elbow dislocation without medial epicondyle fracture [8,29]; one proposed mechanism for this is that rupture of the flexor-pronator origin and ulnar collateral ligament can allow the median nerve to displace posteriorly and into the joint [2].

Under-recognition of the radiographic clues on early follow-up radiographs was an important contributor to delayed diagnoses. All patients in this case series had concentrically reduced joints following closed reduction in the emergency department (Figure 2). However, follow-up radiographs in the three patients with delayed diagnosis all showed tell-tale periosteal ossification in the medial supracondylar ridge, with a radiolucent “lumen” which transmits the median nerve (Figure 3). Radiographic indicators have been previously described in the setting of median nerve entrapment. Matev first reported in 1976 that median nerve entrapment could be recognised radiographically at two to three months post-injury as a cortical depression along the medial distal humerus, now referred to as “Matev’s sign” [12]. Packer and Lennox have subsequently described the “C-sign”, a semicircular defect within the medial epicondylar callus that can be seen at 6 weeks post-injury and represents the entry of the median nerve into a bony tunnel [13]. As these features were present but initially overlooked in all our delayed cases, this underscores the need for greater awareness and education among both orthopaedic surgeons and radiologists to recognise these pathognomonic signs.

US and MRI both have value in the early diagnosis of median nerve entrapment [14,15,16,17]. However, both modalities can be misreported, even by skilled radiologists, as the condition is rare [30]. In our series, false negatives occurred in one of three US and one of four MRIs, contributing to delayed diagnosis. Persistent median nerve dysfunction, even in the setting of a negative US or MRI report, should prompt careful review and consideration of surgical exploration if suspicion remains high.

In our series, the patient with early diagnosis was the only one who achieved complete recovery. Our findings align with Simon [8] and Domingue [7], who reviewed all reported cases of median nerve entrapment post elbow dislocation within the English literature. They observed that only 10 of 34 cases of median nerve entrapment achieved full recovery, all of whom were diagnosed within 4 months of injury. Two of the patients with delayed diagnosis in our series had Matev’s radiographic signs of entrapment present but not recognised within this window for potential nerve recovery (19 and 28 days, Table 1).

In delayed presentations, the optimal surgical management remains unclear. Described treatments within the literature include decompression and neurolysis alone, nerve resection and repair, and nerve resection with reconstruction using autologous nerve grafts–typically sural nerve [3,14]. Importantly, regardless of treatment type, no cases have demonstrated full nerve recovery when surgery was performed more than 4 months post-injury [7,8].

Similarly, Wilks et al. in their study of paediatric supracondylar humerus fractures found that 7% of children presented with median nerve palsy and demonstrated that open reduction led to significantly faster recovery than closed reduction [31]. Although their work addressed fracture-related neuropathy rather than true entrapment, the findings reinforce the principle that median nerve dysfunction in paediatric elbow trauma benefits from timely surgical exploration.

### 4.2. Limitations

This study has several important limitations. Our case series cohort shows significant heterogeneity, particularly in the interval between injury and surgery, which ranged from 4 days to 31 months. These longer intervals represent a substantial developmental window for children, and the unevenly distributed physiological effects of growth and development during this period weaken our comparative analysis. As a retrospective review, neurological examinations and documentation were not standardised. Finally, although this series suggests that early surgical decompression offers the best chance of full recovery, the small numbers and heterogeneity of delayed presentations prevent us from drawing firm conclusions about the optimal surgical strategy for late-diagnosed cases.

### 4.3. Treatment Algorithm

Based on our experience detailed in this report, as well as the existing literature, we propose a hypothetical treatment algorithm based on timing and intraoperative findings (Figure 6).

## 5. Conclusions

Median nerve entrapment following paediatric elbow dislocation is a very rare but serious condition that requires a high index of suspicion and timely action. Although this case series includes a heterogeneous cohort, key takeaways remain to guide assessment and management of this condition. A thorough neurological assessment should be performed at every follow-up point to minimise the risk of a missed diagnosis. Signs of median nerve injury must not be attributed to neurapraxia, and if identified, should be investigated with urgent US or MRI. Clinicians should be aware of the pathognomonic signs of median nerve entrapment that can appear on plain radiographs from 6 weeks post-injury. When diagnosed, median nerve entrapment mandates prompt surgical exploration. Early surgical intervention can lead to complete recovery, while delayed diagnosis and treatment are associated with poorer outcomes.

## Figures and Tables

**Figure 1:**
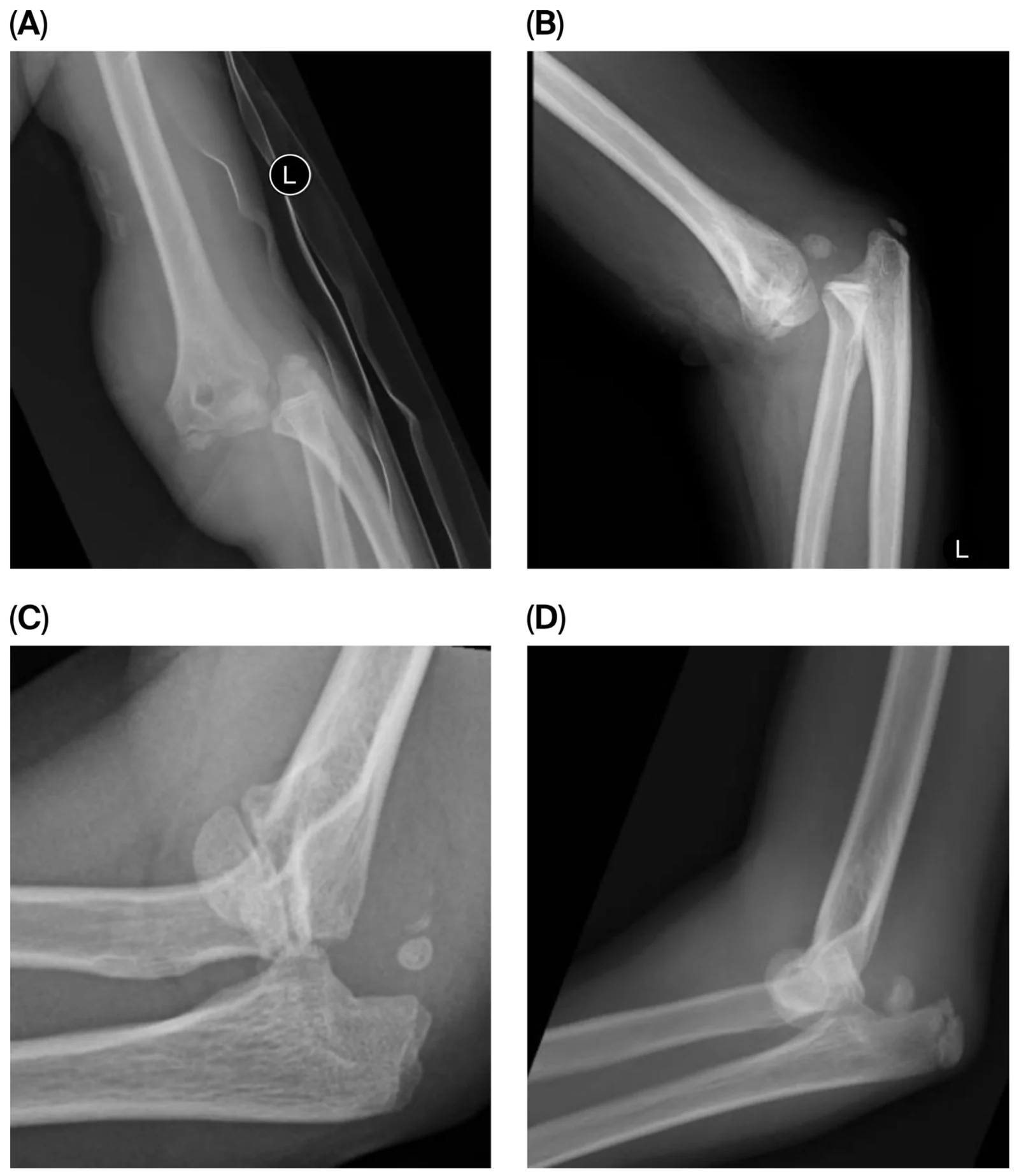
Pre-reduction lateral elbow radiographs ((**A**) Patient 1, (**B**) Patient 2, (**C**) Patient 3, (**D**) Patient 4).

**Figure 2:**
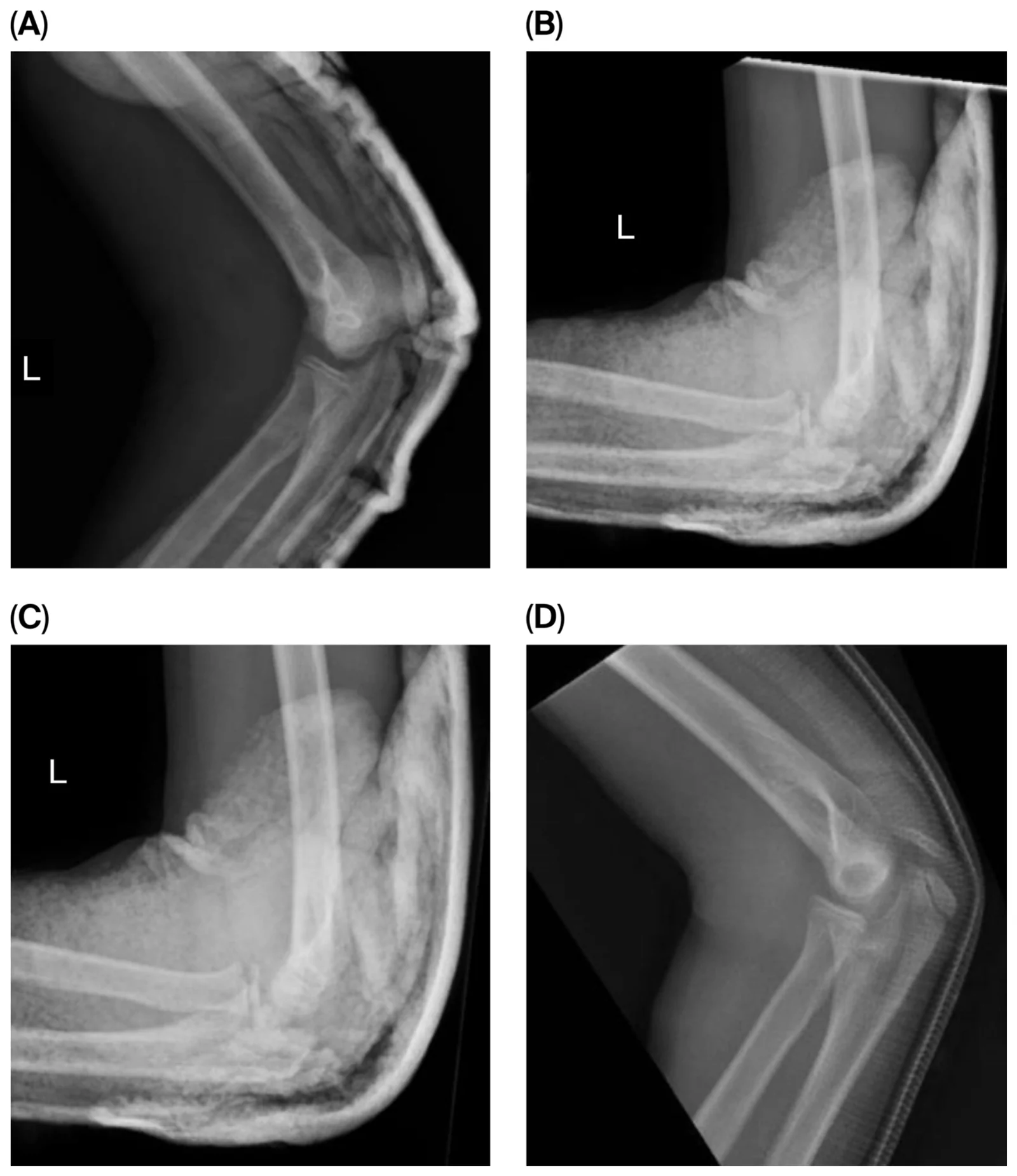
Post-reduction lateral elbow radiographs, demonstrating concentrically reduced joints ((**A**) Patient 1, (**B**) Patient 2, (**C**) Patient 3, (**D**) Patient 4).

**Figure 3:**
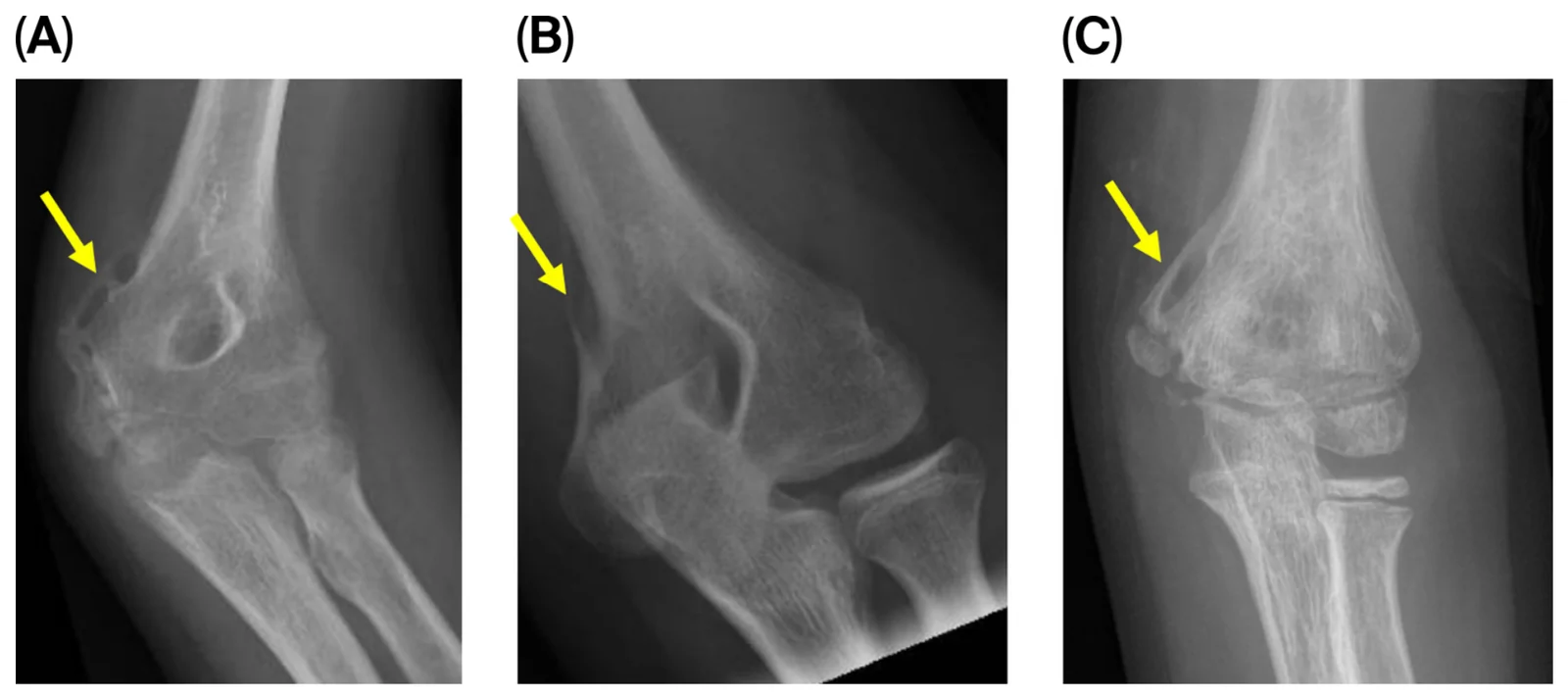
AP elbow radiographs demonstrating bony signs of median nerve entrapment “Matev sign”, with characteristic formation of periosteal bone around the entrapped median nerve. ((**A**) Patient 1 at 2 months post-injury, (**B**) Patient 2 at 29 months post-injury, (**C**) Patient 3 at 3 months post-injury.) Arrow = Matev sign.

**Figure 4:**
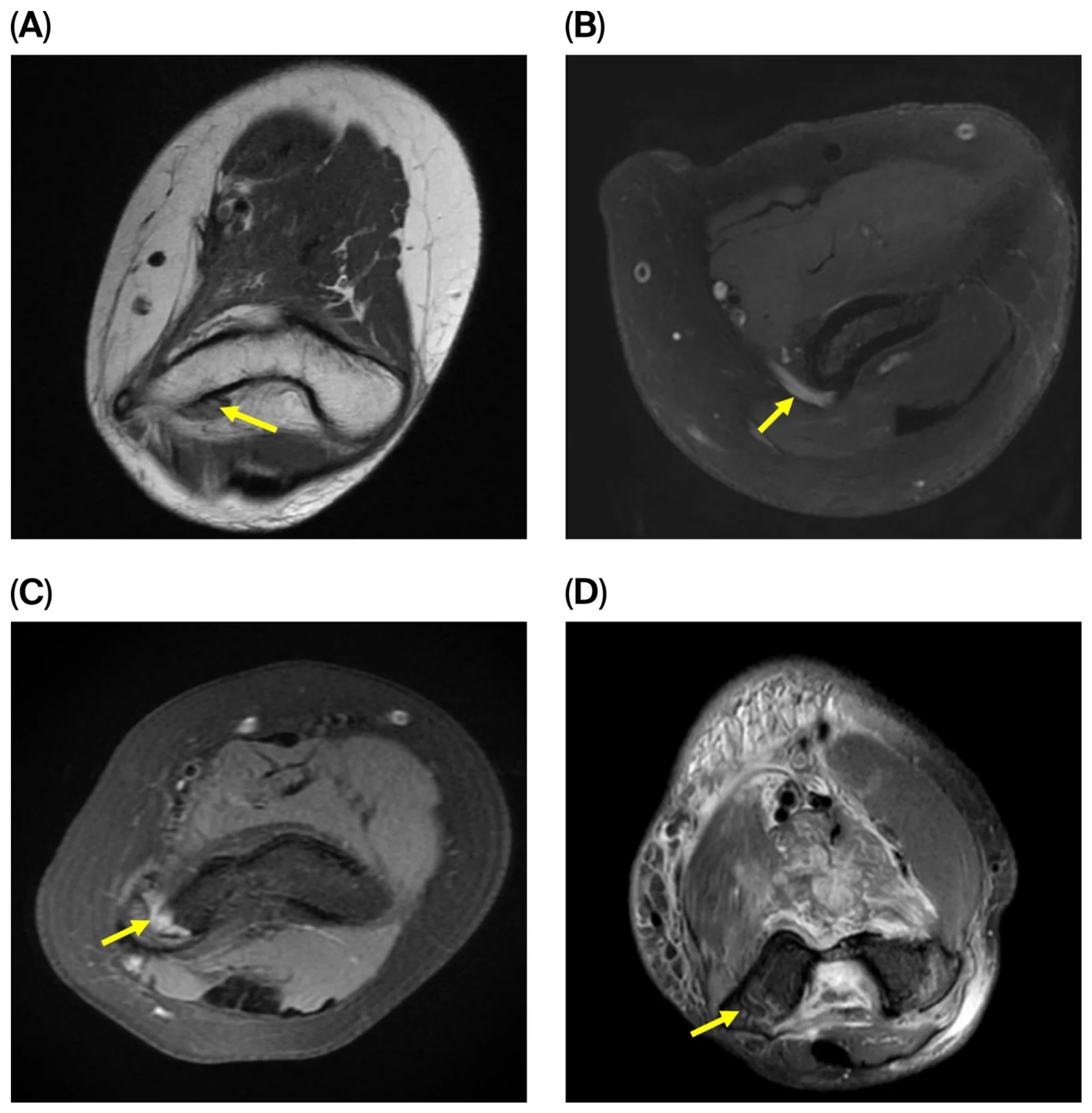
Elbow MRI axial images demonstrating median nerve entrapment. ((**A**) Patient 1, (**B**) Patient 2, (**C**) Patient 3, (**D**) Patient 4.) Arrow = median nerve.

**Figure 5:**
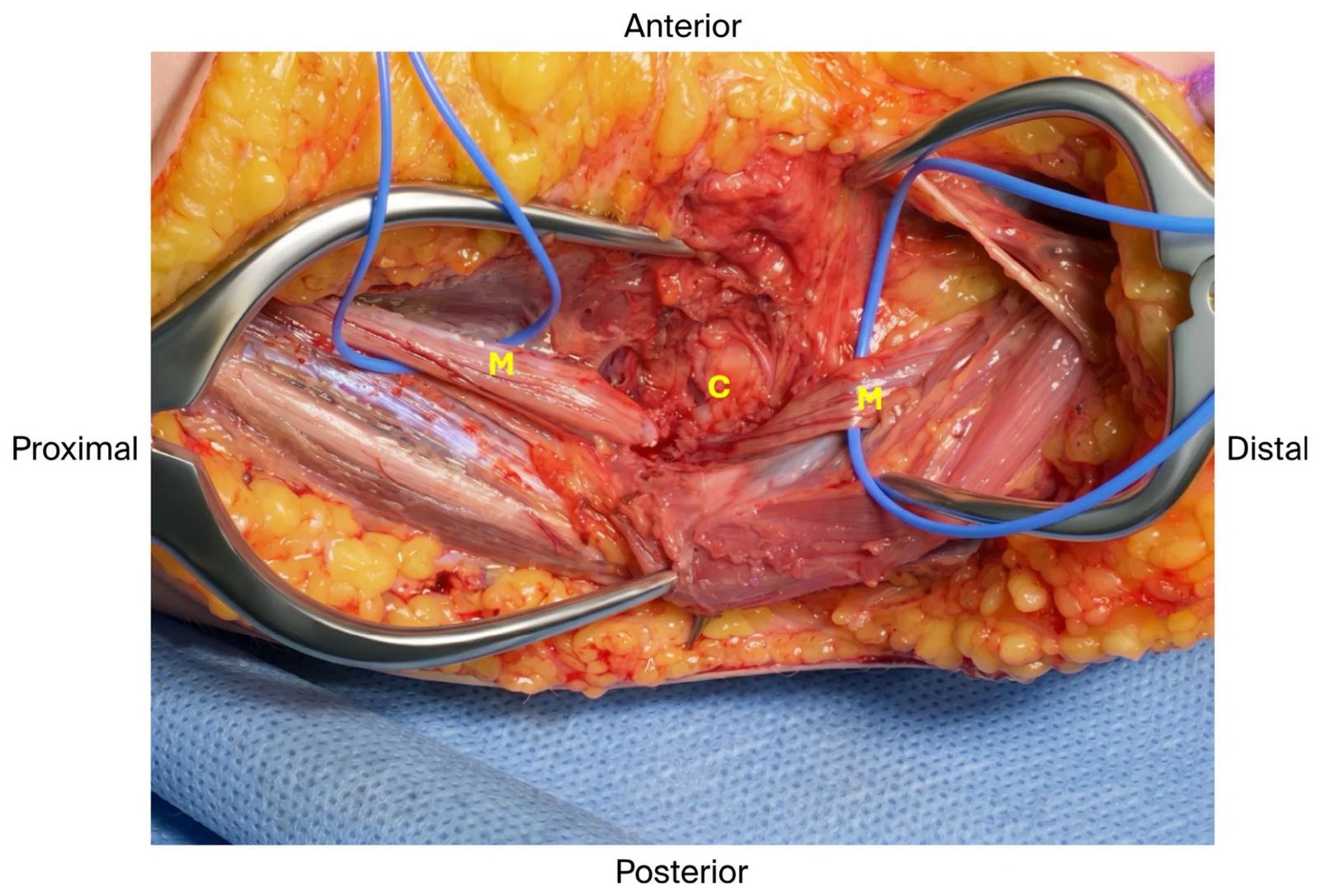
Intra-operative clinical imaging demonstrating median nerve entrapped intra-osseously (Patient 3). M—median nerve, C—callus.

**Figure 6:**
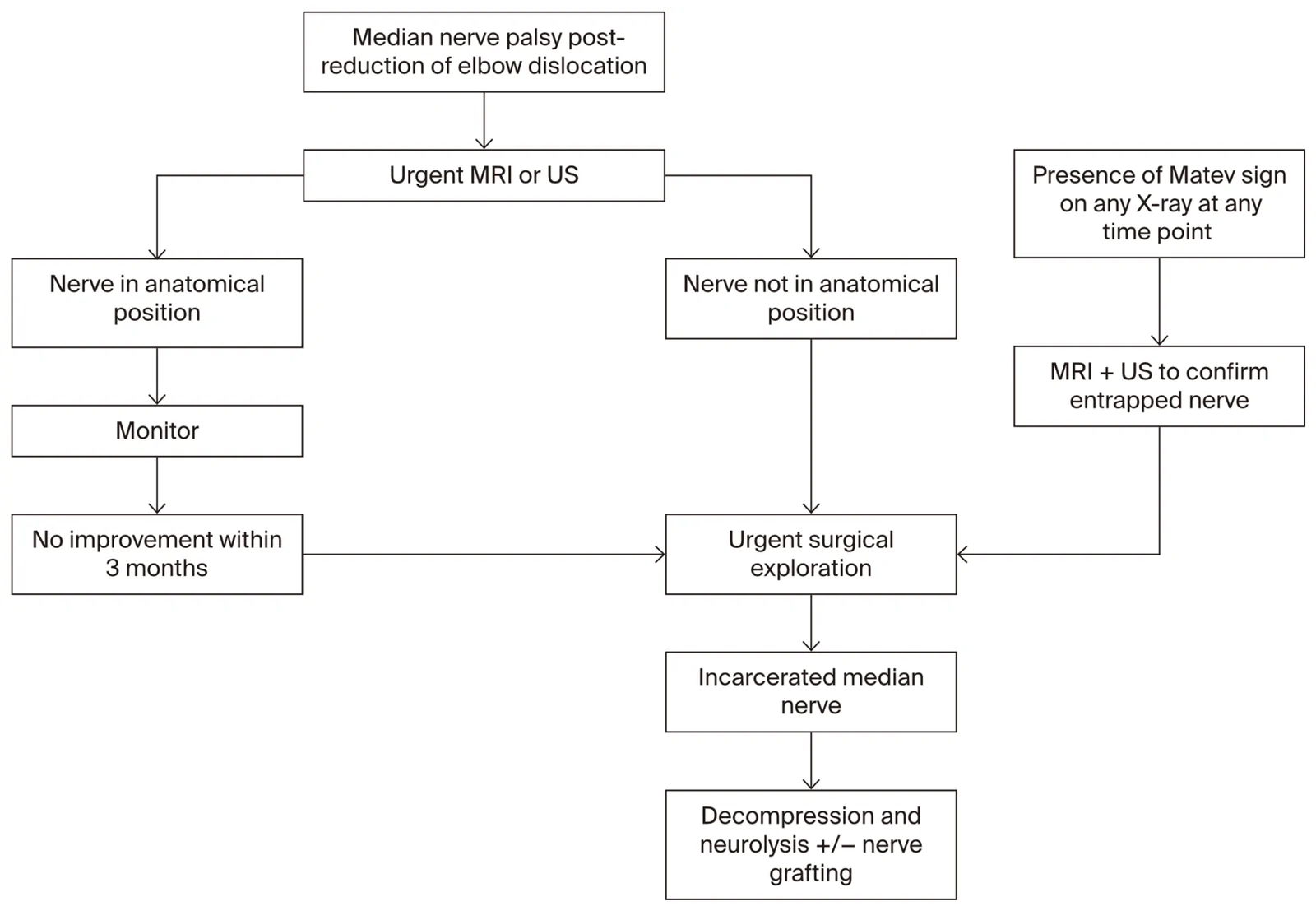
Hypothetical treatment algorithm for investigation and management of elbow dislocations with associated medial epicondyle fractures and signs of median nerve palsy.

**Table 1:** Summary of cases.

Variable	Case 1	Case 2	Case 3	Case 4
Age/Sex	11 M	9 M	9 M	13 F
Dislocation pattern	Posterolateral	Posterolateral	Posterolateral	Posterolateral
Medial epicondyle fracture	Yes	Yes	Yes	Yes
Median nerve deficit post-reduction	Yes	Yes ^a^	Yes	Yes
Imaging				
First radiographic sign of entrapment (days)	28	863	19	N/A ^b^
US evidence of entrapment	Not performed	Yes	No	Yes
Timing of MRI (days post injury)	203	875	228	4
MRI evidence of entrapment	Yes ^c^	Yes	Yes	Yes
Surgery				
Time to surgery (days)	316	934	307	4
Operations performed	(1) Decompression + nerve graft	(1) Decompression (2) Nerve graft	(1) Decompression (2) Nerve graft	(1) Decompression + medial epicondyle fixation
Immediate Pre-Operative Examination				
FPL (MRC)	0	0	0	0
FDP to index finger (MRC)	0	4	3	0
APB (MRC)	Not recorded	Not recorded	0	0
Median nerve sensation	Reduced	Reduced	Reduced	Reduced
Outcomes				
Follow-up (months post-surgery)	66	14	28	9
Complete recovery	No	No	No	Yes
Tendon transfer	No	No	Planned	No
FPL (MRC)	4	1	2	5
FDP to index finger (MRC)	3	4	4	5
APB (MRC)	3	3	4	5
Median nerve sensation	Reduced	Reduced	Reduced	Intact

^a^ Delayed diagnosis of median nerve deficit at 2.5 years post-injury. ^b^ Patient did not have any signs of entrapment on plain radiograph. ^c^ Initial MRI report did not indicate nerve entrapment. FPL = flexor pollicis longus; FDP = flexor digitorum profundus; APB = abductor pollicis brevis; MRC = Medical Research Council Scale for Muscle Strength.

## Data Availability

The data presented in this study are available on request from the corresponding author.

## Abbreviations

The following abbreviations are used in this manuscript:

MRI	Magnetic resonance imaging
US	Ultrasound
